# Association of *Chlamydia trachomatis* infections with preterm delivery; a systematic review and meta-analysis

**DOI:** 10.1186/s12884-018-1868-0

**Published:** 2018-06-18

**Authors:** Amjad Ahmadi, Rashid Ramazanzadeh, Koroush Sayehmiri, Fatemeh Sayehmiri, Nour Amirmozafari

**Affiliations:** 1 Cellular and Molecular Research Center, Research Institute for Health Development, Kurdistan University of Medical Sciences, Pasdaran Street, Sanandaj, 66177-13446 Iran; 2 Microbiology Department, Faculty of Medicine, Kurdistan University of Medical Sciences, Sanandaj, Iran; 3 Psychosocial Injuries Research Center, Ilam University of Medical Sciences, Ilam, Iran; 4 Proteomics Research Center, Shahid Beheshti University of Medical Sciences, Tehran, Iran; 5 Microbiology Department, School of Medicine, Iran University of Medical Sciences, Tehran, Iran

**Keywords:** Preterm delivery, Preterm labor, *Chlamydia trachomatis*

## Abstract

**Background:**

Premature birth is a primary cause of infant mortality and its etiology varies in different countries. *Chlamydia trachomatis (CT)* is a common infectious agent transmitted through sexual contact. The purpose of this study is to investigate the connection between *CT* infections and preterm birth by meta-analysis.

**Methods:**

All articles published in literature databases including Google Scholar, PubMed, ISI (Web of Science), Biological Abs, IranMedex, SID, and Scopus were investigated. Twenty-four relevant articles, authored betweenm 1998–2014 were analyzed through a random effects model. Heterogeneity of the studies was evaluated by I^2^ index. The relationship between years of data collection, sample size, and *CT* infections with preterm delivery prevalence was examined by meta-regression. Data were analyzed with R and STATA [Ver. 12].

**Results:**

The overall prevalence of *CT* infections leading to preterm deliveries was estimated to be 0.13% (CI 95%: 0.11–0.16). The prevalence of *CT* infections leading to preterm deliveries were calculated based on the study method including PCR [0.06 (CI 95%: 0.04–0.09)], serology [0.23 (CI 95%: 0.10–0.35)] and culture [0.17 (CI 95%: 0.10–0.24)]. Analysis indicates that women with chlamydia infections were 2.28 more likely to deliver pre-term in comparison with those who were not infected. It can be concluded that chlamydia infections increase the risks of preterm delivery, OR = 2.28 (95% CI:1.64–3.16).

**Conclusions:**

In regard to the results in numerous studies performed on different continents, this meta- analysis showed a clear association between preterm delivery and prior *CT* colonization*.*

**Electronic supplementary material:**

The online version of this article (10.1186/s12884-018-1868-0) contains supplementary material, which is available to authorized users.

## Background

*Chlamydia trachomatis* (*CT*) is a gram-negative non-motile bacterium, 0.2–1.4 μm in size, that tend to live inside the cylindrical cells of human epithelium [1]. It is the most common infection transmitted through sexual intercourse and those infected may unconsciously transmit it to their sexual partners [2]. In terms of clinical manifestations, colonization with this bacterium by in large is asymptomatic (80% of cases). Patients often do not seek medical treatment up until the symptoms become prominent [3]. Studies have shown that about four million people in the world are infected with *CT* [4].

Premature birth is a primary cause of infant mortality and the costs of caring for preterm infants are often high. Complications of preterm birth include hyaline membrane disease, necrotizing enterocolitis, air leak syndrome, and intraventricular hemorrhage of the brain. If the new-born survives, they may face convulsions, hearing disorders, or visual disturbances [5]. Rates of premature births vary in different societies, 5–15% in North America and 1.3% in Iran [6, 7].

Different factors can lead to the occurrence of preterm births including heart disease, multiple birth, repeated abortions, diabetes, and genitourinary tract infections. Genitourinary infections may be responsible for 25–40% of preterm births. These infections are caused by bacteria such as *Mycoplasma urealyticum (MU), Mycoplasma huminis (MH), Neisseria gonorrhea (NG), Trichomonas vaginalis (T. vaginalis)*, and *Chlamydia trachomatis* (*CT*). The role of *CT* infections in preterm birth has not been well established [8]. Several studies have been conducted on the outcome of *CT* infections [9–31]. The aim of this investigation is to evaluate the results of these studies by meta-analysis.

## Methods

### Literature identification

**Fig. 1:**
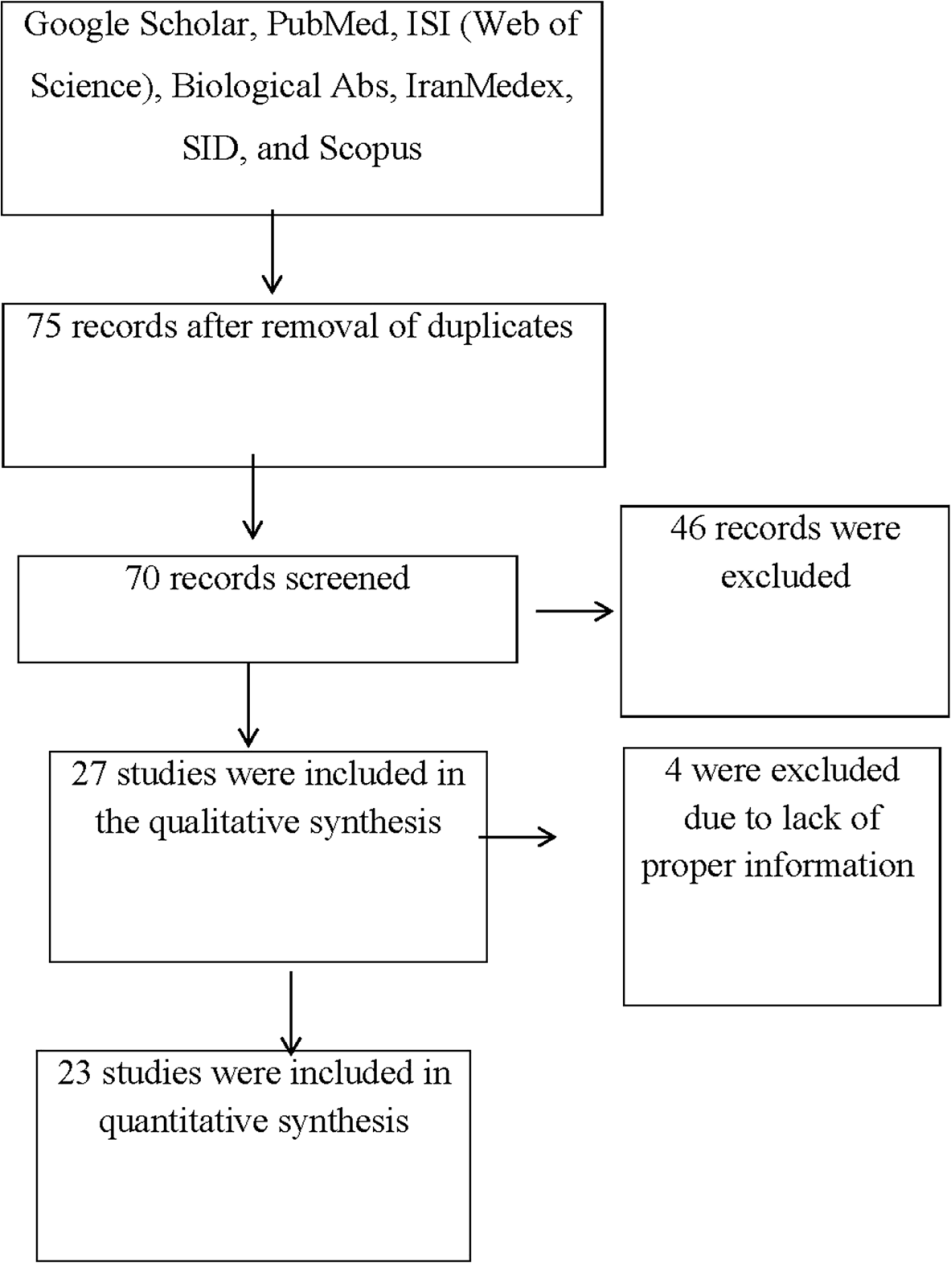
The study flowchart of selected articles for final analysis

We used PRISMA guidelines for this article [32]. Our data were based on articles that were published in local and international journals. We conducted the literature searches with the following keywords: *CT* and preterm delivery, *CT* and preterm labor, *CT* and preterm birth, *CT* and premature delivery, *CT* and prematurity, and *CT* and premature child birth. All medical literature published in databases including Google Scholar, PubMed, ISI (Web of Science), Biological Abs, IranMedex, SID, and Scopus between, 1986–2014 were chosen. In the first search, 75 manuscripts published between, 1986–2014 were selected. We excluded all duplicate articles, which left 23 for analysis (Fig. 1) [33, 34].

### Inclusion and exclusion criteria

All papers with the keywords from above in the title or the abstract were included in our first list and irrelevant articles were removed from the study. The search process was performed by two persons and adjusted one by one. The most important biases in meta-analysis are: publication bias and selection bias. Publication bias was checked using the Begg’s test. We reduced the possibility of selection bias by clearly defining the criteria for data collection in each selected study by two researchers independently. These selected studies became the final list.

### Data extraction

An information checklist for research papers consisted of first author’s last name, year of publication, country where the study was carried out, mean age of participants, sample size, study period, method of bacteria identification, the type of study, and existence of a significant to no significant relationship between *CT* infections with preterm birth. Studies were excluded if they presented insufficient data, if they were mere reviews, or if they were not epidemiologic studies.

### Statistical analysis

Variance of prevalence for CT in each study were calculated as binomial distributions. Studies were combined based on their sample sizes and variance of samples. Due to the heterogeneity of the studies, the random effects model was used when combining studies. To assess heterogeneity, the Cochrane Q test and I^2^ statistics were used. A *p*-value less than 5% was considered a significant heterogeneity test. To examine publication bias, the Begg’s test and funnel plot was used. A funnel plot is a graphical detection of publication bias that is actually a bivariate scatter plot (*x, y*) of sample size versus estimates of effect size.

Subgroup analysis was done according to a diagnostic method, type of study, and continents. Meta-regression was used to explore relationship between prevalence of *CT* associated with preterm delivery including the year of the study and sample size. To find association between Chlamydia infections and the risk of preterm delivery, an odds ratio (OR) was used and statistical analyses were performed in STATA version 11.1 and metan commend.

## Results

**Fig. 2:**
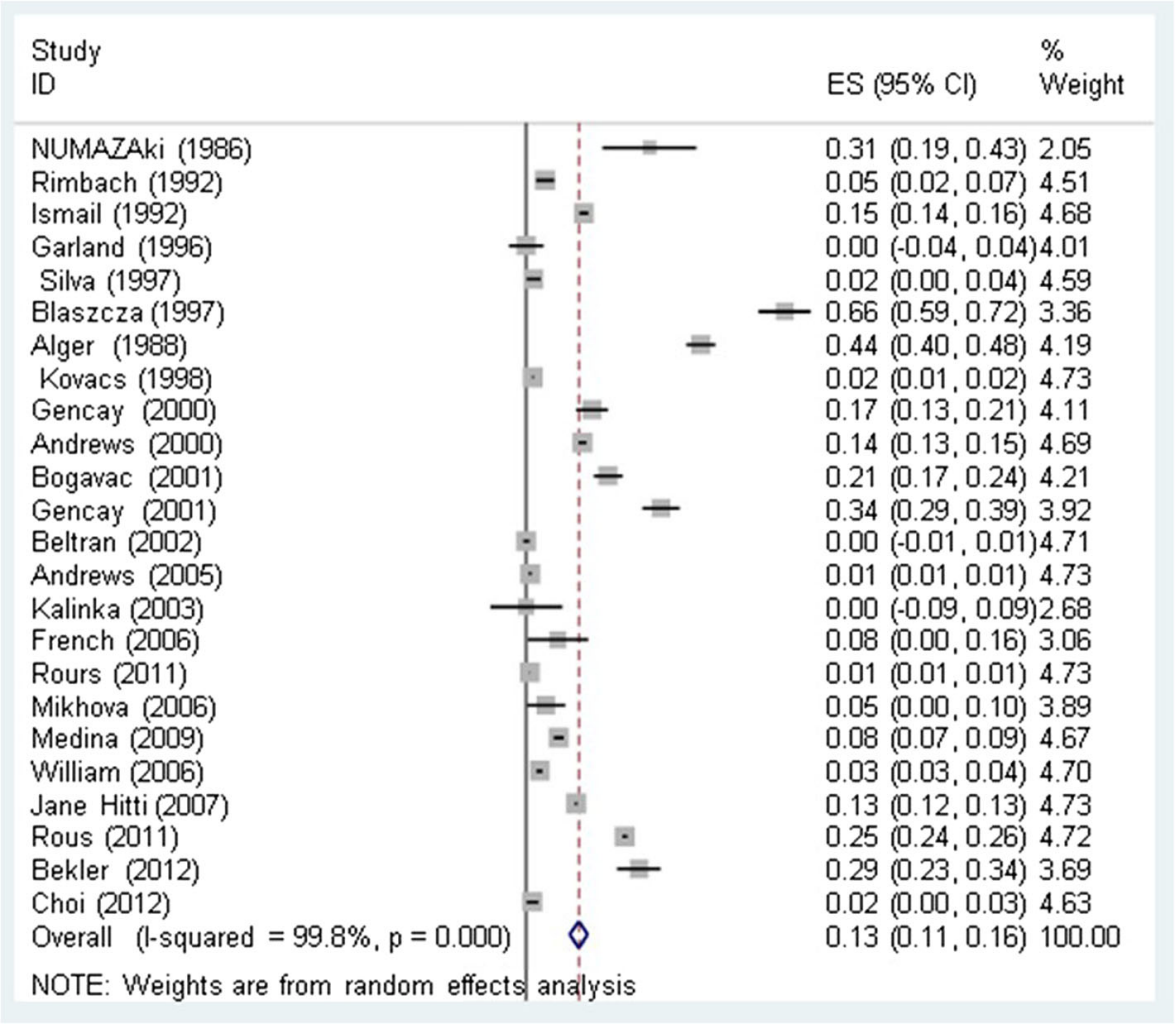
Prevalence of *CT* infections with preterm births and its 95% confidence interval using a random-effect model. Midpoint of each line segment represents the estimated prevalence in the study. Rhombic mark shows the prevalence in Total, extracted from all studies

We used 23 relevant studies (1986–2014) were included in this meta-analysis (Additional file 1.). The overall relatedness of *CT* infections with preterm delivery was estimated to be 0.13% (CI 95%: 0.11–0.16) (Fig. 2). The prevalence of *CT* infections with preterm delivery based on the study method including PCR [0.06 (CI 95%: 0.04–0.09)], serology [0.23 (CI 95%: 0.10–0.35)], and culture [0.17 (CI 95%: 0.10–0.24)]. The prevalence of *CT* infections leading to preterm deliveries based on study type including case-control [0.16 (CI 95%: 0.11–0.21)] and cross-sectional [0.13 (CI 95%: 0.08–0.17)] was determined. The prevalence of *CT* infections leading to preterm delivery based on geographical location including Europe [0.13 (95% CI: 0.09–0.18)], Asia [0.08 (CI 95%: –0.01–0.17)], the USA [0.15 (CI 95%: 0.08–0.22)], and Africa [0.29 (CI 95%: 0.23–0.34)] were estimated (Table 1). Analysis showed that pregnant women with chlamydia infections were 2.28 times more likely to deliver prematurely in comparison to those who were not infected.

**Table 1:** Prevalence of *CT* infection with preterm delivery according to different factors

		Study NO.	Prevalence^a^ (95% CI)	Weight %
Diagnostic method	PCR	10	0.06 (0.04–0.09)	45.50
	Serology	8	0.23 (0.10–0.35)	27.90
	Culture	6	0.17 (0.10–0.24)	26.60
	Total	24	0.13 (0.11–0.24)	100
Study type	Case – control	9	0.16 (0.11–0.21)	36.67
	Sectional	15	0.13 (0.08–0.17)	66.33
	Total	24	0.13 (0.11–0.24)	100
*Continent*	Europe	14	0.13 (0.09–0.18)	59.55
	Asia	3	0.08 (−0.01–0.17)	10.70
	USA	6	0.15 (0.08–0.22)	20.06
	Africa	1	0.29 (0.23–0.34)	3.69
	Total	24	0.13 (0.11–0.24)	100
Recognize group	NO	14	0.04 (0.02–0.05)	57.08
	Yes	10	0.26 (0.19–0.33)	42.92
	Total	24	0.13 (0.11–0.24)	100

^a^Point estimate and confidence interval were estimated using random effects model

**Fig. 3:**
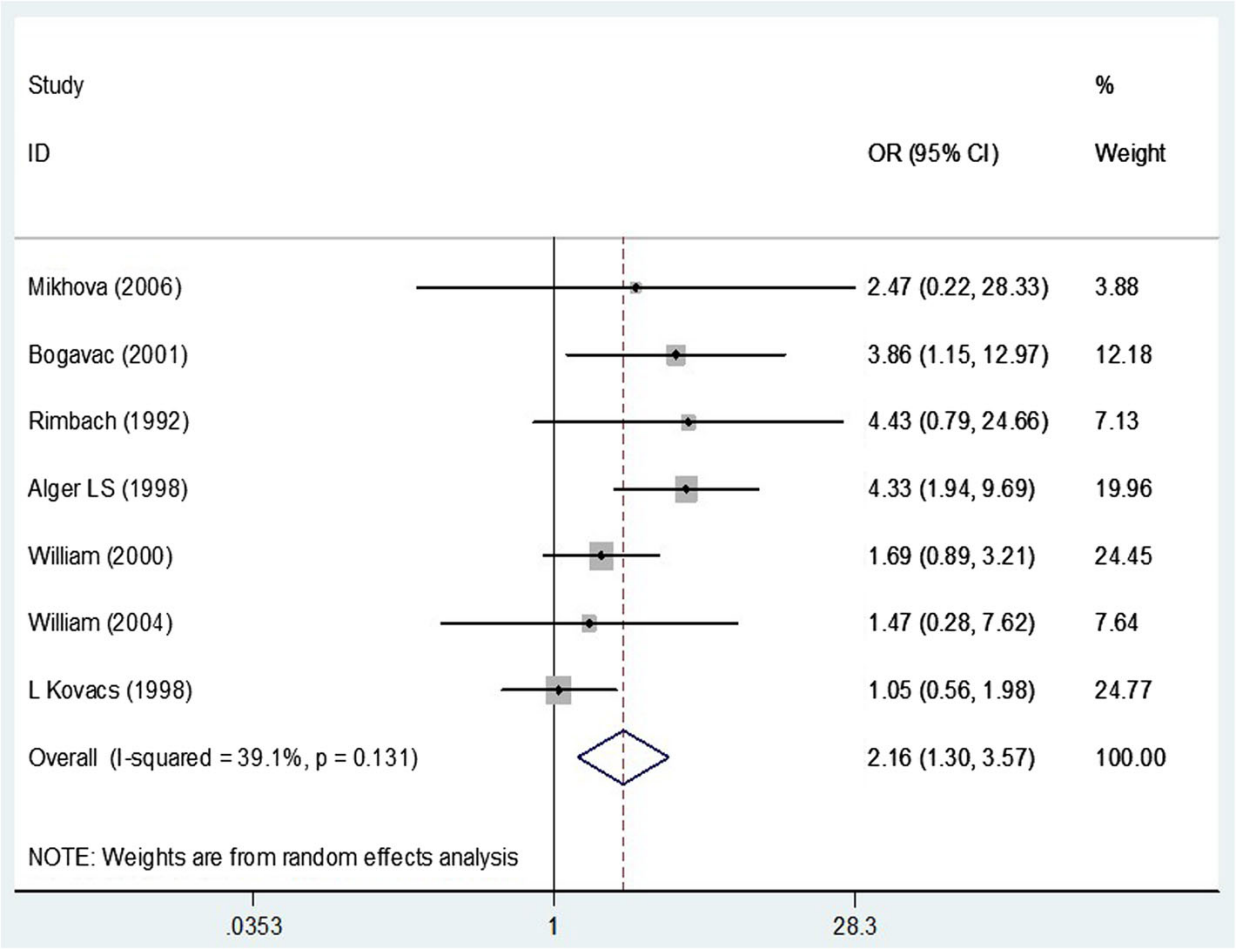
Meta-analysis of the association of *CT* infections with preterm delivery risk. Odds Ratio and 95% confidence intervals for each study and in summary with weighting in a random-effect model are shown. Midpoint of each line segment represents the estimated OR in the study. Rhombic mark shows the OR in Total, extracted from all studies

We can conclude that chlamydia colonization increased the risk of premature labor, OR = 2.16(CI 95%: 1.3–3.57) (Fig. 3). For OR analysis, random effect models were used. I^2^ was significant (*p* = 0.013).

**Fig. 4:**
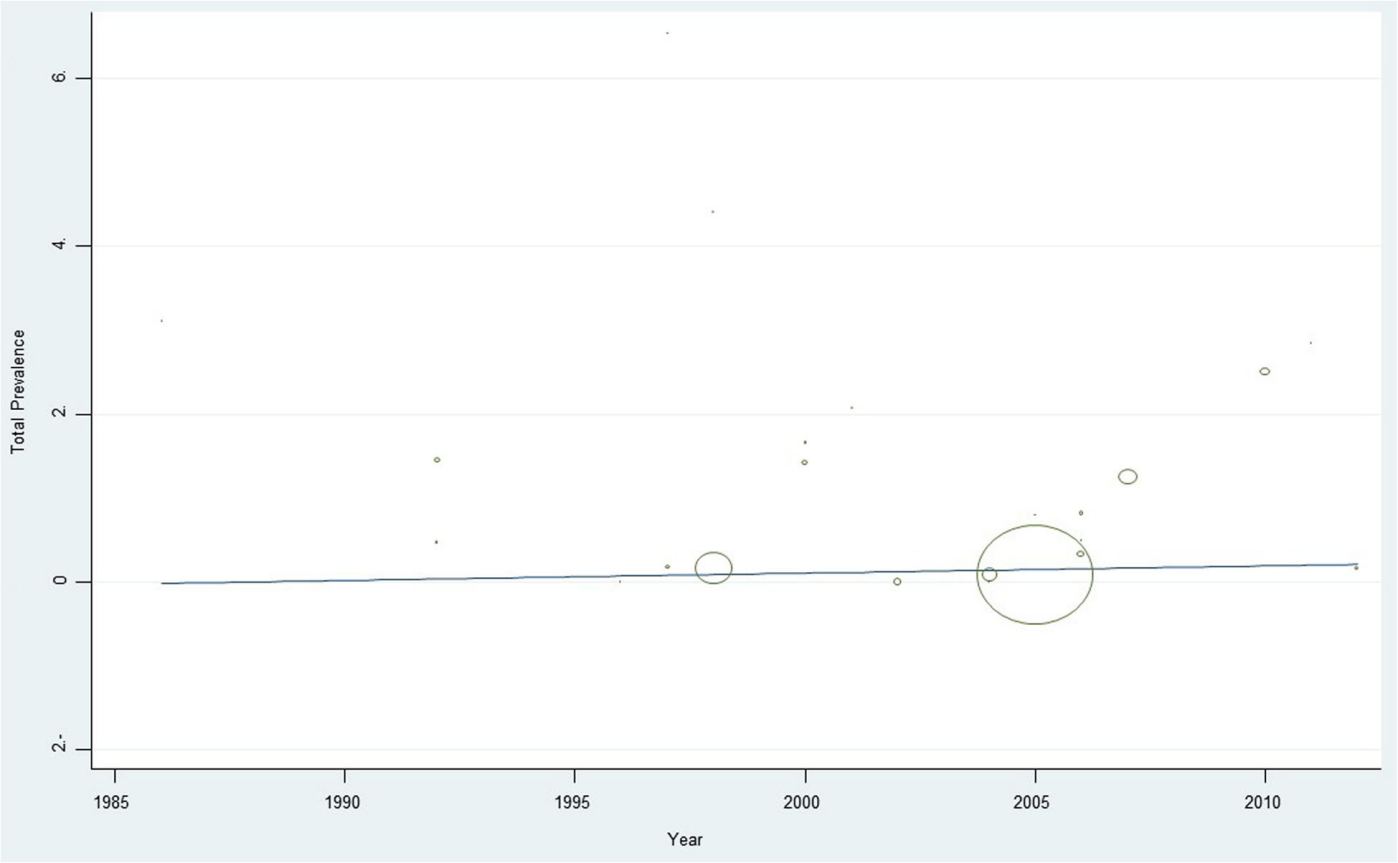
Meta-regression of *CT* infections with preterm delivery prevalence with year of data collection as variable

**Fig. 5:**
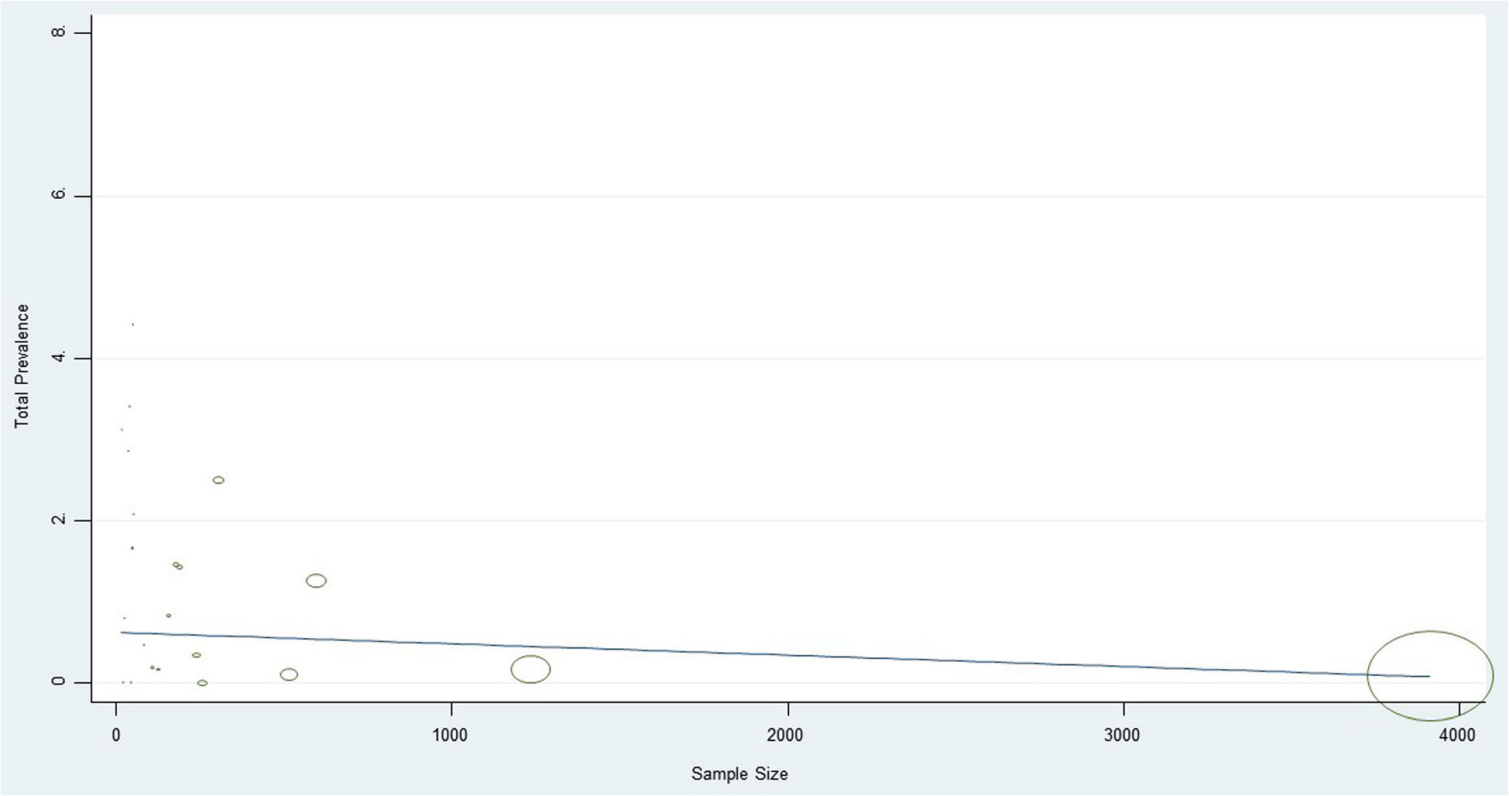
Meta-regression of *CT* infections with preterm delivery prevalence with sample size as variable

Our interpretation of the meta-regression data showed that there was no significant relationship between the prevalence of *CT* and preterm delivery with the year of study (*p* = 0.33) and sample size (*p* = 0.21) (Figs 4 and 5).

**Fig. 6:**
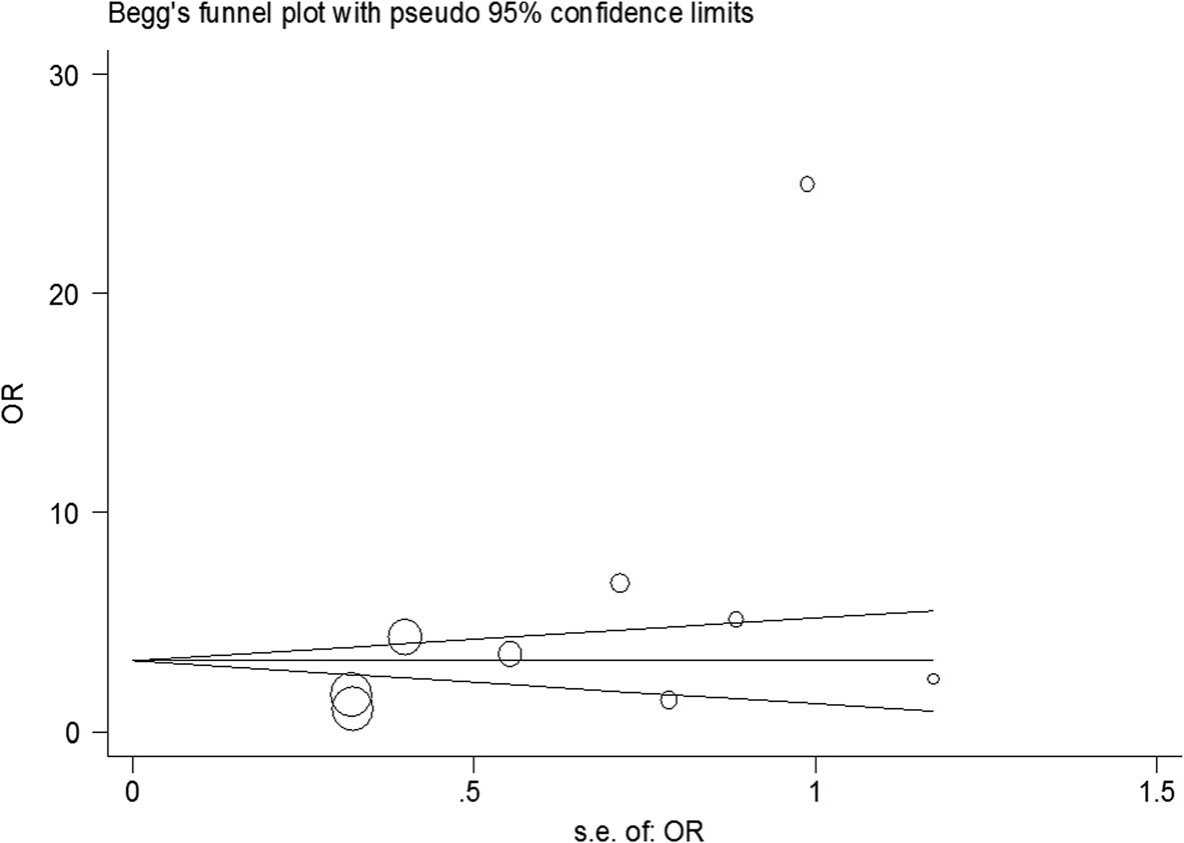
Begg’s funnel plot for publication bias in the risk difference (RD) analysis. The diameter of each circle represents the weight in the meta–analysis

According to our analysis as publication bias, there were no significant biases in these studies. In fact, most studies were located inside our funnel plot and the results were included in our analysis (Fig. 6).

## Discussion

*Chlamydia trachomatis* is an intracellular pathogen and immunologic response to *CT* infection elaborate T cell response and cytokine release. Because inflammatory reactions induce damage in tissue, preterm birth may be the consequence of those inflammatory responses [17, 18, 25, 30, 35]. In this meta-analysis, it turned out that there is a significant relationship between *CT* infections and preterm delivery. Our interpretation of the meta-regression showed that there was no significant relationship between prevalence of *CT* infections and preterm delivery with the year of study (*p* = 0.33) and sample size (*p* = 0.21). The prevalence of *CT* infections and preterm delivery in Europe and America was relatively high. Among the articles that were studied, we noted some contradictory results. In Mikhova et al. (2006) using PCR methodology found that there was no relationship between *CT* infections and preterm birth observed [26]. Another PCR study by William et al. (2000) showed that there was a connection between *CT* infection and preterm birth [18]. In a cross sectional study using culture technique, Ismail et al. (1992) reported a meaningful correlation between *Chlamydia trachomatis* infections and preterm birth [10]. In another cross sectional study by Silva et al. (1997) using bacterial cultures observed no correlation between *CT* infection and preterm birth [12]. Bogavac et al. (2001) showed that a significant relationship between the *CT* infection and preterm birth was observed in a case-control study using serology [19]. Rimbach et al. (1992) using the same method, showed no connection between preterm birth and *Chlamydia trachomatis* infections [9]. In regard to the overall results based on studies performed on diverse populations, this meta- analysis showed a clear association between preterm delivery and prior colonization with *Chlamydia trachomati*s.

## Conclusions

In regard to the results of numerous studies performed on different continents, this meta-analysis showed a clear association between preterm delivery and prior *CT* colonization*.*

## Additional file

Additional file 1: Summary of the included studies evaluating the prevalence of *CT* infections with preterm deliveries. (XLSX 14 kb)

## Abbreviations

CT: Chlamydia trachomatis
MH: Mycoplasma huminis
MU: Mycoplasma urealyticum
NG: Neisseria gonorrhea
TV: Trichomonas vaginal
